# The Effect of Scan Length on the Assessment of BOLD Delay in Ischemic Stroke

**DOI:** 10.3389/fneur.2020.00381

**Published:** 2020-05-05

**Authors:** Ayse Ceren Tanrıtanır, Kersten Villringer, Ivana Galinovic, Ulrike Grittner, Evgeniya Kirilina, Jochen B. Fiebach, Arno Villringer, Ahmed A. Khalil

**Affiliations:** ^1^Center for Stroke Research, Charité - Universitätsmedizin Berlin, Berlin, Germany; ^2^Institute of Biometry and Clinical Epidemiology, Charité – Universitätsmedizin Berlin, Berlin, Germany; ^3^Berlin Institute of Health (BIH), Berlin, Germany; ^4^Department of Neurophysics, Max Planck Institute for Human Cognitive and Brain Sciences, Leipzig, Germany; ^5^Center for Cognitive Neuroscience Berlin, Free University, Berlin, Germany; ^6^Berlin School of Mind and Brain, Humboldt Universität zu Berlin, Berlin, Germany; ^7^Department of Neurology, Max Planck Institute for Human Cognitive and Brain Sciences, Leipzig, Germany

**Keywords:** perfusion, acute stroke, BOLD delay, scan length, MRI

## Abstract

**Objectives:** To evaluate the impact of resting-state functional MRI scan length on the diagnostic accuracy, image quality and lesion volume estimation of BOLD delay maps used for brain perfusion assessment in acute ischemic stroke.

**Methods:** Sixty-three acute ischemic stroke patients received a 340 s resting-state functional MRI within 24 h of stroke symptom onset. BOLD delay maps were calculated from the full scan and four shortened versions (68 s, 136 s, 204 s, 272 s). The BOLD delay lesions on these maps were compared in terms of spatial overlap and volumetric agreement with the lesions derived from the full scans and with time-to-maximum (Tmax) lesions derived from DSC-MRI in a subset of patients (n = 10). In addition, the interpretability and quality of these maps were compared across different scan lengths using mixed models.

**Results:** Shortened BOLD delay scans showed a small volumetric bias (ranging from 0.05 to 5.3 mL; between a 0.13% volumetric underestimation and a 7.7% overestimation relative to the mean of the volumes, depending on scan length) compared to the full scan. Decreased scan length was associated with decreased spatial overlap with both the BOLD delay lesions derived from the full scans and with Tmax lesions. Only the two shortest scan lengths (68 and 136 s) were associated with substantially decreased interpretability, decreased structure clarity, and increased noisiness of BOLD delay maps.

**Conclusions:** BOLD delay maps derived from resting-state fMRI scans lasting 272 and 204 s provide sufficient diagnostic quality and adequate assessment of perfusion lesion volumes. Such shortened scans may be helpful in situations where quick clinical decisions need to be made.

## Introduction

The assessment of brain perfusion in acute ischemic stroke can be used to guide clinical decision-making, particularly when considering the use of intravenous thrombolysis or mechanical thrombectomy in patients otherwise ineligible for these treatments (1–3). Dynamic susceptibility contrast MRI (DSC-MRI) is most commonly used for this purpose in routine clinical practice. However, it requires the use of exogenous contrast agents, which have potentially severe side effects (4). Even in people with normal kidney function, the gadolinium-based contrast agents used for DSC-MRI accumulate in the brain with repeated administration (5, 6), which has led the European Medicines Agency to recommend restricting their use (7).

As part of the ongoing search for alternative perfusion imaging methods, many studies have recently shown that the temporal properties of the blood-oxygenation-level-dependent (BOLD) signal reflect aspects of perfusion. This is because the BOLD signal, while usually used to probe the hemodynamic response to neural activity, reflects an amalgam of different physiological processes. These include fluctuations originating from outside the brain that travel through the vasculature in a manner closely resembling blood flow (8–11). These non-neuronal oscillations, referred to as systemic low-frequency oscillations (sLFOs), exist in the low-frequency range of the signal (0.01–0.15 Hz). Studies have shown changes in the amplitude, frequency, and phase of sLFOs in areas of low blood flow (12–15). Currently, one of the most well-studied temporal properties of the BOLD signal in relation to perfusion is BOLD delay (also known as hemodynamic lag)–the delay in arrival of sLFOs to a certain voxel compared to a reference region (14).

Evidence for the relationship between BOLD delay and perfusion comes from several sources. Firstly, regions of BOLD delay have been detected in cerebrovascular disorders including stroke (14, 16–21), transient ischemic attack (22), and Moyamoya disease (23). Such regions are also observed in other conditions associated with more subtle changes in cerebral perfusion such as Alzheimer's disease (24), epilepsy (25), and sickle cell disease (26). Secondly, direct comparisons between perfusion modalities have shown that BOLD delay correlates with measures of perfusion derived from DSC-MRI (14, 16, 18–21, 27) and arterial spin labeling (17, 22, 23). In addition, we have recently shown that increased BOLD delay in acute stroke due to large vessel occlusion is reversible following vessel recanalization, and that this reversibility mirrors reperfusion detected using DSC-MRI (19). Thirdly, manipulation of the circulatory system through respiratory challenges affects sLFOs in a manner consistent with changes in cerebrovascular reactivity (28–34). Finally, the physiological basis for BOLD delay seems to lie in axial variations in the concentration of deoxyhemoglobin within arteries and veins caused by vasomotion or changes in oxygen saturation (35). These variations act as a “virtual tracer”—a contrast agent intrinsic to the blood (35).

All this evidence suggests that the use of BOLD delay for assessing perfusion is a promising alternative to DSC-MRI. However, the clinical applicability of BOLD delay is still limited due to its relatively long acquisition time, which particularly hampers its use in clinical situations where decisions have to be made extremely quickly, such as in acute stroke. Studies utilizing BOLD delay for brain perfusion assessment have varied widely in scan length, between 3.5 and 30 min (see Table 1 for an overview), markedly longer than typical DSC-MRI scans (~2 min). In this study, we evaluated the impact of scan length on the diagnostic accuracy, image quality and lesion volume estimation of BOLD delay maps used for brain perfusion assessment in acute ischemic stroke.

**Table 1:** Overview of resting-state functional MRI scan lengths used in previous studies on BOLD delay for the assessment of brain perfusion.

**Study**	**Investigated population**	**Scan length (min:s)**
Lv et al. (14)	Acute stroke	5:50
Amemiya et al. (16)	Acute stroke and chronic hypoperfusion	10:00
Qian et al. (36)	Acute stroke	10:00
Christen et al. (23)	Moyamoya disease	3:36
Coloigner et al. (26)	Sickle cell disease	6:00
Siegel et al. (17)	Subacute stroke	30:00^a^
Qian et al. (37)	Healthy individuals	6:40
Khalil et al. (18)	Acute stroke	5:50
Ni et al. (20)	Subacute stroke	8:00
Wu et al. (38)	Chronic hypoperfusion and moyamoya disease	5:00^b^
Tong et al. (27)	Healthy individuals	6:00
Chen et al. (21)	Acute stroke	8:00
Yang et al. (39)	Healthy individuals	10:00
Lv et al. (22)	Transient ischemic attack	8:00
Yan et al. (24)	Alzheimer's disease, mild cognitive impairment, and healthy individuals	6:40
Khalil et al. (19)	Acute stroke	5:50
Zhao et al. (40)	Chronic stroke	6:15
Nishida et al. (32)	Chronic hypoperfusion and moyamoya disease	7:20
Jahanian et al. (41)	Moyamoya disease and healthy individuals	4:00 to 6:00

a *Minimum of 5 min of resting-state functional MRI data after motion scrubbing required*.

b *Total resting-state functional MRI data acquired was 20 min, but data was divided into pre- and post-acetazolamide administration (5 min each) for processing*.

## Materials and Methods

### Study Design

Patients with a confirmed clinical and radiological diagnosis of ischemic stroke who received a resting-state functional MRI scan together with a standard stroke protocol were recruited as part of the Longitudinal MRI Examinations of Patients With Brain Ischemia and Blood-Brain Barrier Permeability [LOBI-BBB] study clinicaltrials.gov from June 2016 to December 2017. The LOBI-BBB study is a single-center, prospective cohort study of patients with acute ischemic stroke. A subset of patients received a follow-up (day 1) scan within 24 h of the first scan session (day 0 scan). This study was approved by the local institutional review board (EA1/200/13) and only patients who gave written informed consent were included. No exclusion criteria based on head motion were used, because we aimed at achieving a representative clinical population in our sample.

### Imaging Protocol

A standard stroke MRI protocol was performed on a Siemens (Erlangen, Germany) Tim Trio 3 Tesla MRI scanner. The imaging protocol included a T2^*^-weighted image (TR/TE=669/20 ms, matrix = 320 × 320, field of view = 220 mm, slice thickness = 5 mm, acquisition time = 1 min 21 s), a diffusion-weighted image (TR/TE = 8900/93 ms, matrix = 192 × 192, field of view = 229 mm, slice thickness = 2.5 mm, acquisition time = 2 min 21 s), a FLAIR image (TR/TE = 8000/96 ms, matrix = 232 × 256, field of view = 199^*^220 mm, slice thickness = 5 mm, acquisition time = 1 min 21 s) and a time-of-flight MR angiography (TR/TE = 21/3.4 ms, matrix = 218 × 384, field of view = 162 × 199 mm, slice thickness = 0.5 mm, acquisition time = 3 min 2 s). In addition, a resting-state functional MRI was performed using a multiband EPI scan (University of Minnesota sequence cmrr_mbep2d_bold R008) (42, 43): [850 (resting state) time points, TR/TE = 400/30 ms, flip angle 43°, matrix = 64 × 64, 192 × 192 mm field of view (FOV), multiband factor = 6, thirty 4.0 mm thick slices, acquisition time: 340 s]. During the scan, patients were requested to relax, lie still, and close their eyes. In a subset of patients, DSC-MRI data were acquired following the injection of a bolus of 5 mL Gadovist 1 mol/L and a saline flush at a flow rate of 5 mL/s with the following scanning parameters: TR/TE = 1390/29 ms, flip angle = 60, matrix = 128 × 128, 21 slices, slice thickness = 5 mm, acquisition time = 1 min 58 s.

### Image Processing

#### Preprocessing

As a first step, the first 25 volumes (10 s) were removed from the full 340 s resting-state scan to allow for magnetization equilibrium. Then, four shortened sets of data of various lengths were produced from the resulting full 330 s resting state scan (68 s, 136 s, 204 s, 272 s), and are referred to in this paper as 0.2, 0.4, 0.6, 0.8 scan segments, respectively.

Preprocessing was performed on the resting-state data of differing lengths using FSL (https://fsl.fmrib.ox.ac.uk/fsl) and AFNI (https://afni.nimh.nih.gov/afni). This comprised volume realignment to the first volume, regression of the effect of three rigid body translations and three rotations, spatial smoothing with 6-mm Gaussian kernel, and band pass filtering (0.01–0.15 Hz). The mean framewise displacement across the scan segments (FD) was calculated (44) and rescaled (multiplied by 10) to increase the comparability of this variable with the rest of the coefficients in the mixed effects models. In addition, to assess the signal change related to head motion, DVARS was calculated, defined as the frame-to-frame root mean square change in voxel intensities averaged across the entire brain (44).

#### Time Shift Analysis

A template was used to extract the reference time series by averaging the BOLD signal across all voxels in the major venous sinuses. This template was created from the post-gadolinium high-resolution T1-weighted scans of eight subjects, as described in the Supplemental Material of (18). Briefly, segmented gray matter, white matter, and CSF tissue masks were combined and subtracted from the brain-extracted T1 image, leaving an image containing only the vessels. This was registered to MNI space, binarized, summed up across subjects, manually edited to remove voxels outside the major venous sinuses, and spatially smoothed.

The venous sinus was preferred over the whole brain signal because BOLD delay calculated using the venous sinus reference correlated more strongly with DSC-MRI-based perfusion maps in both chronic cerebrovascular disease (23) and acute stroke (18). The venous sinus reference also avoids the contamination of the BOLD delay assessment procedure by hypoperfused voxels because it extracts time courses from voxels outside the brain parenchyma.

BOLD delay maps were generated by assigning each voxel the value of the time shift that achieves maximum cross-correlation between the reference time series and the voxel's time series. For this purpose, we used *rapidtide*, which is a set of Python tools for finding time-lagged correlations (https://github.com/bbfrederick/rapidtide). Significance thresholds for the cross-correlation were set by *rapidtide* using a shuffling procedure (10,000 times) to calculate the distribution of null correlation values. To determine the offset associated with the highest correlation coefficient with the reference signal, we shifted the time course from −20 to +20 s. This long tracking range was necessary because delays in acute stroke patients have been shown to be very long (18, 19).

BOLD delay maps generated from the data of each scan segment were registered to an echo-planar imaging (EPI) template derived from a similar cohort of stroke patients (18, 19). A multi-stage registration procedure (rigid → affine → deformable) was applied using the *antsRegistrationSyn* script from the ANTs software (http://stnava.github.io/ANTs/) (45). Lesions on the diffusion-weighted images (DWI) were manually delineated by a stroke researcher (A.C.T.) and checked by a senior stroke researcher and radiology resident (A.K.) The DWI and the delineated DWI lesions were registered to the template using ANTs. DWI lesions were masked during the registration process to improve registration (46). The results of all registrations were inspected visually for quality.

### Quantitative Analysis

Patients with a visible perfusion lesion on their full-length BOLD delay maps (assessed by A.K., a stroke researcher and radiology resident with 7 years' experience with perfusion imaging in stroke) were eligible for quantitative analysis in this study. Perfusion lesions on the BOLD delay maps were automatically delineated using an in-house algorithm used in a previous study (19). This algorithm searches the vascular territory affected by the stroke (defined as the vascular territory where the acute DWI lesion is present) for areas of hypoperfusion on the BOLD delay maps. For this procedure, BOLD delay maps thresholded to >0 s, >2.3 s, and >4.6 s were analyzed separately, generating three perfusion lesion volume values for each dataset (one value per threshold). The potential influence of these thresholds on the outcomes of this study were accounted for in the models.

#### Spatial Comparison of BOLD Delay Lesions

The Dice similarity coefficient was used for spatially comparing perfusion lesions on BOLD delay maps from shorter scans with those from the full length scan. The Dice similarity coefficient is a statistic used for evaluating spatial overlap and ranges between +1 (perfect overlap) and 0 (no overlap) (47). The association between scan length and the Dice similarity coefficient value was investigated using a linear mixed model (two-level; random intercept), with head motion as a covariate (48).

#### Volumetric Comparison of BOLD Delay Lesions

BOLD delay lesion volumes were calculated and the following analyses were performed on them:

Bland-Altman analysis (49) was used for assessing the agreement between shorter scans and the full length scan on BOLD delay lesion volume.

A linear mixed effects model (two-level; random intercept and slope model) was used to investigate the association between scan length and BOLD delay lesion volume. Since the distribution of BOLD delay lesion volumes was skewed, the volumes were log-transformed for this analysis. The BOLD delay thresholds (0 s, 2.3 s, 4.6 s; ref = 0 s) and the scan sessions (day 0 or day 1; ref = day 0) were accounted for in this model.

#### Spatial and Volumetric Comparison of a Subsample of Patients With DSC-MRI Data

In a subsample of the patients eligible for quantitative analysis (*n* = 10), DSC-MRI data was acquired during the same scanning session after the rsfMRI scan. DSC-MRI data were analyzed using Stroketool version 2.8 (2011 Digital Image Solutions—HJ Wittsack) by selecting an arterial input function of 5–10 voxels in the middle cerebral artery contralateral to the acute infarction (50). Time-to-maximum (Tmax) maps were calculated using block-circulant singular value decomposition deconvolution of the concentration-time curve (51).

In this subsample, the Tmax maps were delineated using the same automated procedure described above for the BOLD delay maps, after applying a Tmax threshold of >6 s (18, 52, 53). The Dice similarity coefficient was calculated between the perfusion lesions derived from each of the BOLD delay scans of different lengths and the Tmax maps. A Bland-Altman analysis was also performed to assess the agreement between Tmax perfusion lesion volumes and BOLD delay perfusion lesion volumes derived from the scans of different lengths.

### Qualitative Analysis

Patients were included for qualitative analysis if they met the study's inclusion criteria, regardless of whether or not there was a visible perfusion lesion on their BOLD delay maps.

Two radiologists [K.V. [rater 1] and I.G. [rater 2]], both experienced in stroke perfusion imaging, visually assessed the BOLD delay maps of different scan lengths. The raters were blinded to all patient data and to the length of the scans from which the BOLD delay maps were generated, but had access to the DWI corresponding to each BOLD delay map. Prior to performing the readings, the raters were shown examples of the maps derived from an independent dataset (see Supplementary Figure 2 for examples) and trained on how to fill in the data entry sheet.

#### Interpretability of Shortened BOLD Delay Maps

The raters assessed whether or not a perfusion lesion was visible on the BOLD delay maps, or if the BOLD delay map was uninterpretable. We calculated the agreement between the BOLD delay maps derived from each of the shortened scans and the BOLD delay maps derived from the full scan using unweighted Cohen's kappa. A binary logistic mixed model (48) (two level; random-intercept) was executed for comparing the interpretability of the BOLD delay maps (reference = “uninterpretable”) derived from scans of different lengths while accounting for head motion and raters (ref = “rater 1”).

#### Quality of BOLD Delay Maps

The raters assessed how noisy the BOLD delay maps were (on a scale of 1 to 3, with 3 indicating the highest level of noise) and how clear certain structures such as the ventricles were on the map (on a scale of 1 to 3, with 3 indicating the highest structure clarity). Examples of maps of various noisiness and structure clarity are shown in Supplementary Figure 2.

We used the quadratic-weighted Cohen's kappa (54) to assess the agreement between raters on the interpretability, noisiness, and structure clarity of the BOLD delay maps derived from each scan length.

Ordinal mixed models (55) were used to investigate the association between scan length and noise as well as structure clarity of the maps (two-level; random intercept models).

Note that in all statistical models used in this paper, subjects were level two units such that intra-individual correlation among the measures collected on a particular individual was taken into account and scan session identification (reference = “day 0”) was included as a covariate in the models. All models in the qualitative analysis also accounted for the influence of the raters (reference = “rater 1”).

### Statistical Analysis

All statistical analyses were performed using *R* Statistical Software (56). The data and the code used for statistical analysis and data visualization in this study are publicly available at https://github.com/ahmedaak/BD_scan-shortening. Bland-Altman analysis was performed using the R package “blandr” (57), metrics of inter-rater agreement were calculated using the R package “irr” (58), linear mixed models using the “lmer” and “glmer” functions from the R package “lme4” (48), and ordinal mixed models using the “clmm” function from the R package “ordinal” (55). The distribution of continuous variables in different groups is visualized in this paper using raincloud plots, which combine dot plots, box plots, and violin plots (59). The distribution of categorical variables in different groups is visualized using spine plots (60).

## Results

**Figure 1:**
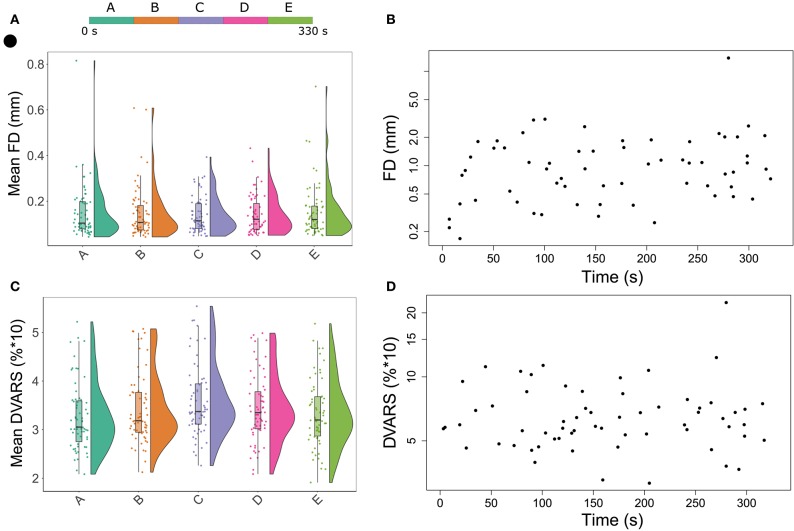
Raincloud plots showing the distribution of mean head motion metrics (framewise displacement in **(A)** DVARS in **(C)** for each segment of the resting-state functional MRI scan. In Figures 1B,D, each point represents the maximum head motion value across the full scan (from one patient) measured using framewise displacement **(B)** and DVARS **(D)**, plotted against time.

Sixty-three patients who underwent an MRI scan within 24 h of stroke symptom onset were eligible for qualitative analysis. Forty-three of these patients had perfusion lesions on their BOLD delay maps and were selected for quantitative analysis. The characteristics of the two study groups for quantitative and qualitative analysis are presented separately in Table 2. The amount of head motion in each part of the rsfMRI scan is depicted for all patients in Figure 1.

**Table 2:** Demographics and clinical characteristics of the study sample.

**Variable**	**Whole sample**	**Patients with hypoperfusion**
N	63	43
Age in years (median, IQR)	75 (65–79)	78 (65–83)
Sex (M/F)	39/24	23/20
Follow-up (*n*)	38	17
**mRS (median, IQR)**		
Admission	3 (2–4)	4 (3–4)
Discharge	2 (1–3)	3 (1–4)
**NIHSS (median, IQR)**		
Admission	4 (1–8)	7 (3–11)
Discharge	2 (0–3)	3 (1–5)
Previous stroke (*n*)	22	16
Time (in hours) from symptom onset to MRI (median, IQR)	9 (3–16)	8 (1–14)
Vessel occlusion on MRA (n)	26	24
**Therapy (*****n*****)**		
Thrombolysis	17	15
Mechanical thrombectomy	5	5
**Stroke vascular territory (*****n*****)**		
Anterior cerebral artery	1	0
Middle cerebral artery	29	19
Posterior cerebral artery	9	3
Multiple territories	24	21

*MRS, Modified Rankin Scale; NIHSS, National Institutes of Health Stroke Scale*.

The characteristics of the subsample who received a DSC-MRI scan are as follows: median age = 77.5 years (IQR = 63.5–80.3 years), median mRS at admission = 4 (IQR = 4–5), median mRS at discharge = 3 (IQR = 1–4), median NIHSS at admission = 11 (IQR = 7–16), median NIHSS at discharge = 4 (IQR = 2–8), median time from symptom onset to imaging = 1.5 h (IQR = 1–6.5 h). In this subsample, 7/10 patients were female, 3/10 had a follow-up MRI the next day, 4/10 had a previous stroke, 8/10 had a vessel occlusion on the TOF-MRA, 7/10 received intravenous thrombolysis, 4/10 received mechanical thrombectomy, 9/10 had an infarct in the MCA territory, and 1/10 had an infarct in the PCA territory. Data processing took a mean of 122 s (0.2 scan), 193 s (0.4 scan), 273 s (0.6 scan), 368 s (0.8 scan), and 411 s (full scan) per patient on an Intel® Xeon® X5570 CPU (2.93 GHz, 4 cores) with 64 GB of RAM. Note that a single thread was used for the processing.

### Quantitative Analysis

The DWIs and BOLD delay maps of all the patients in the quantitative analysis sample can be found here: https://doi.org/10.6084/m9.figshare.12022728.

#### Spatial Comparison of BOLD Delay Lesions

**Figure 2:**
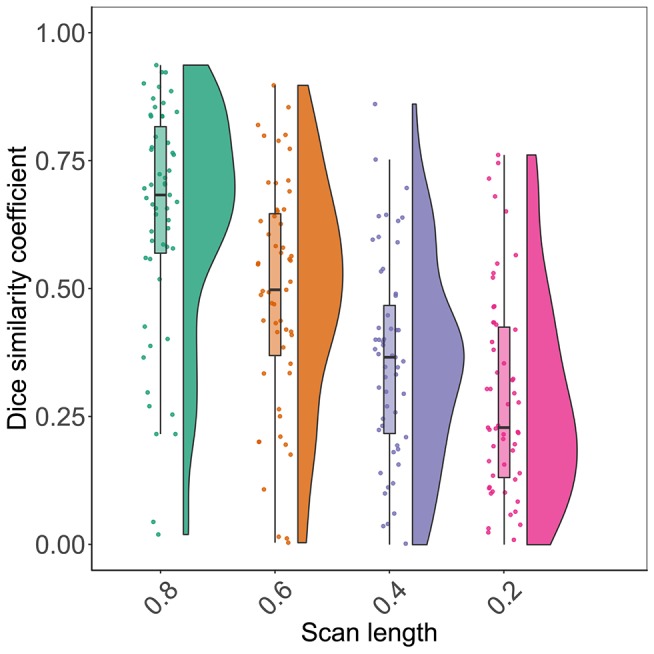
Raincloud plots showing the distribution of Dice similarity coefficients (y axis) representing the degree of spatial overlap between BOLD delay lesions derived from resting-state functional MRI scans of different lengths (x axis) and the BOLD delay lesions derived from the full scan.

Figure 2 shows the distribution of spatial overlap metrics (Dice similarity coefficients) between BOLD delay lesions from each shortened scan and BOLD delay lesions from the full scan. The highest spatial overlap was between the 0.8 scan and the full scan (median = 0.68; IQR=0.56–0.81) and it decreased with decreasing scan length. The median Dice similarity coefficients between perfusion lesions from each BOLD delay scan length and Tmax perfusion lesions (for the subsample who also received DSC-MRI) were as follows: full scan = 0.29 (IQR = 0.02–0.33), 0.8 scan = 0.26 (IQR = 0.03–0.40), 0.6 scan = 0.17 (IQR = 0.03–0.39), 0.4 scan = 0.1 (IQR = 0.02–0.29), 0.2 scan = 0.16 (IQR = 0.02–0.32).

The results of the linear mixed model of the association between scan length and spatial overlap showed that a 1% decrease in scan length was associated with a 0.006 reduction on average in the Dice coefficient between the BOLD delay lesions derived from the shortened scan and the full scan (beta = 0.006, SE = 0.0004, *t* = 13.6, *p* < 0.0001).

#### Volumetric Comparison of BOLD Delay Lesions

**Figure 3:**
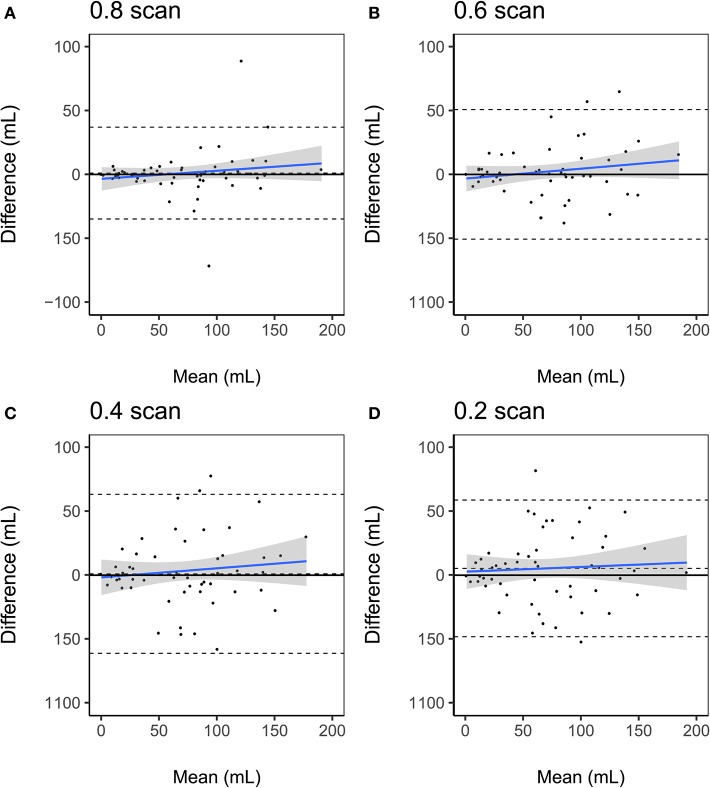
Bland-Altman plots of volumetric agreement between BOLD delay lesion volumes derived from resting-state functional MRI scans of different lengths **(A)** 0.8 scan, **(B)** 0.6 scan, **(C)** 0.4 scan, **(D)** 0.2 scan and BOLD delay lesion volumes derived from the full scans. The upper and lower dashed lines represent the 95% limits of agreement and the middle dashed lines represent the bias (mean difference). The blue solid line and shaded gray region represent the regression lines and 95% confidence interval of the regression lines, respectively.

The results of the Bland-Altman analysis are presented in Figure 3 and the distribution of lesion volumes for each scan length is shown in Supplementary Figure 1. Compared to the BOLD delay lesion volumes derived from the full scan, the biases of the lesion volumes derived from the shortened scans were as follows: the 0.2 scan = 5.3 mL (a 7.7% overestimation relative to the mean of both scans, limits of agreement = −48.1–58.7 mL), 0.4 scan = 1.04 mL (a 4.5% overestimation relative to the mean of both methods, limits of agreement = −61.1–63.2), 0.6 scan = 0.05 mL (a 0.13% underestimation relative to the mean of both methods, limits of agreement = −50.6–50.7 mL), and 0.8 scan = 1.06 mL (a 1.4% overestimation relative to the mean of both methods, limits of agreement = −34.8–36.9 mL).

The results of the Bland-Altman analysis for the subsample who also received a DSC-MRI scan are shown in Supplementary Figure 3. Compared to the Tmax perfusion lesion volumes, the biases of the BOLD delay lesion volumes were as follows: the 0.2 scan = −14.5 mL (a 68.1% underestimation relative to the mean of both methods [BOLD delay and Tmax], limits of agreement = −80.6–51.5 mL), 0.4 scan = −8.2 mL (a 36.7% underestimation relative to the mean of both methods, limits of agreement = −68.0–51.7 mL), 0.6 scan = −18.3 mL (a 43.7% underestimation relative to the mean of both methods, limits of agreement = −80.2–43.5 mL), 0.8 scan = −17.2 (a 56.3% underestimation relative to the mean of both methods, limits of agreement = −65.7 to 31.3 mL), full scan = −12.9 mL (a 58.6% underestimation relative to the mean of both methods, limits of agreement = −58.6–32.6 mL).

**Figure 4:**
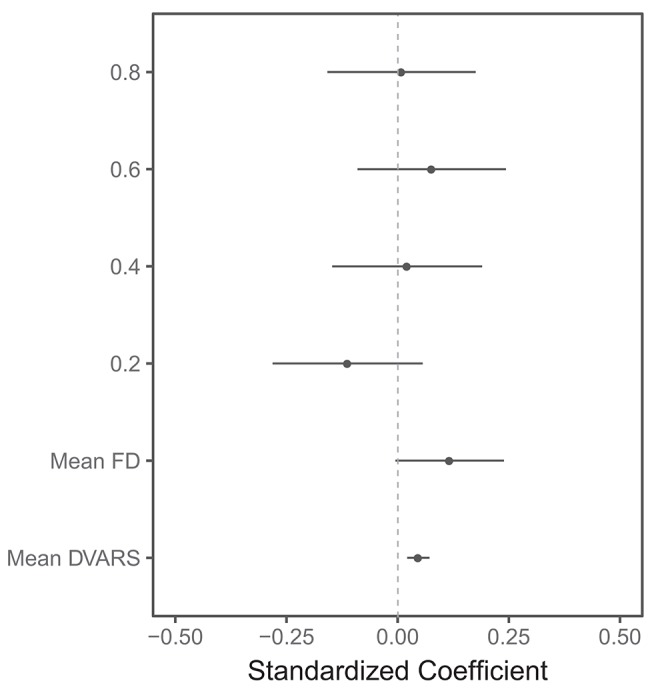
Visualization of the results of the linear mixed model of the association between several predictors and BOLD delay lesion volumes. The points represent the standardized (beta) coefficients and the lines represent the 95% confidence intervals of the coefficients for each predictor. FD: framewise displacement. The scan lengths are shown as 0.8, 0.6, 0.4, and 0.2 with the full scan used as the reference category. This plot shows that the head motion metrics (mean FD and mean DVARS) are significantly associated with larger BOLD delay lesion volumes. There is no statistically significant difference between BOLD delay lesion volumes derived from the shorter resting-state functional MRI scans and the full scan. Note that only a subset of the predictors in this model are shown here—the numerical results of this linear mixed model, including the rest of the predictors, are shown in Supplementary Table 1.

The linear mixed model showed that there was no systematic impact of scan length on lesion volumes (Figure 4 and Supplementary Table 1). Head motion measured using mean DVARS was associated with larger BOLD delay lesion volumes (beta = 0.05, 95% CI = 0.02–0.07, *t* = 3.64, *p* = 0.0003).

### Qualitative Analysis

#### Diagnostic Accuracy

**Figure 5:**
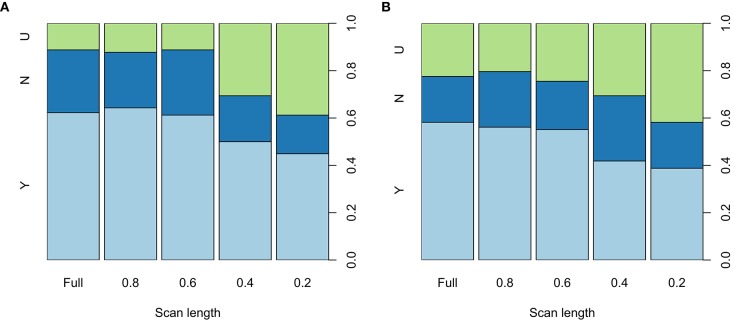
Spine plots showing the distribution of the qualitative ratings of the BOLD delay maps derived from different resting-state functional MRI scan lengths made by rater 1 **(A)** and 2 **(B)** The maps were rated as either showing a perfusion lesion (Y, light blue), not showing a perfusion lesion (N, blue), or being uninterpretable (U, green). Statistically significant differences in interpretability are seen between the full scan and the 0.4 and 0.2 shortened scans. The quantitative results of the binary logistic mixed model investigating the effects of scan length on the interpretability of the BOLD delay maps are shown in Supplementary Table 3.

The interpretability of the BOLD delay maps derived from different scan lengths is shown in Figure 5 for each of the raters. Agreement on map interpretability between BOLD delay maps derived from different scan lengths and those derived from the full scan are shown for each rater in Supplementary Table 2. The binary logistic mixed model revealed that scan lengths 0.2 (odds ratio = 0.21, 95% CI = 0.12–0.37, *p* < 0.0001) and 0.4 (odds ratio = 0.37, 95% CI = 0.21–0.64, *p* = 0.0004) were associated with decreased interpretability of the BOLD delay maps. Longer scans (0.8 and 0.6) showed no substantial association with interpretability of the BOLD delay maps (Supplementary Table 3).

#### Inter-Rater Agreement

Table 3 shows the inter-rater agreement on the interpretation, noisiness, and structure clarity of the BOLD delay maps derived from different scan lengths. The raters' agreement on the interpretation of the BOLD delay maps was good across scan lengths (Cohen's kappa 0.64–0.82). Agreement on noisiness and structure clarity was markedly higher in the BOLD delay maps derived from the shorter scans than in those derived from the longer scans.

**Table 3:** Inter-rater agreement (quadratic-weighted Cohen's kappa) on BOLD delay maps derived from different scan lengths.

**Scan length**	**Interpretation^**a**^**	**Noise^**b**^**	**Structure clarity^**b**^**
Full	0.64	0.22	0.40
0.8	0.71	0.29	0.15
0.6	0.82	0.25	0.26
0.4	0.75	0.67	0.40
0.2	0.67	0.72	0.48

a *Refers to whether the rater judged the map as showing aperfusion lesion, not showing a perfusion lesion, or being uninterpretable*.

b *Judged as high, medium or low*.

#### Quality of BOLD Delay Maps

**Figure 6:**
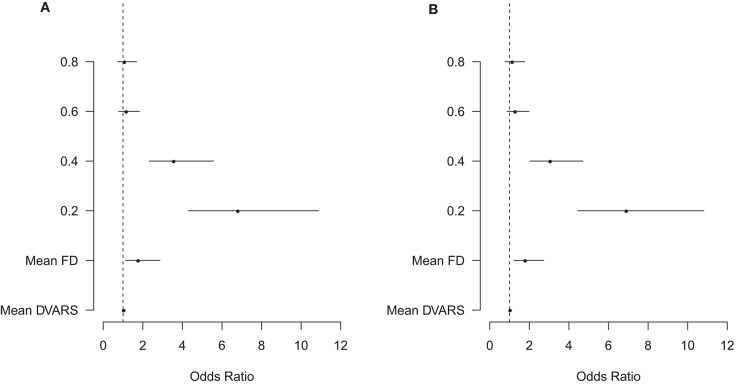
Visualization of the results of the ordinal mixed models of the association between several predictors and BOLD delay map noisiness **(A)** and structure clarity **(B)**. The points represent the odds ratios and the lines represent the 95% confidence intervals of the odds ratios for each predictor. The vertical dashed line represents an odds ratio of 1. FD, framewise displacement. The scan lengths are shown as 0.8, 0.6, 0.4, and 0.2 with the full scan used as the reference category. The plot shows that head motion (mean FD and mean DVARS) as well as the shortened 0.4 and 0.2 scans are associated with a significantly higher odds of more noise and less structure clarity than full scans. Note that only a subset of the predictors in this model are shown here—the numerical results of this ordinal mixed model, including the rest of the predictors, are shown in Supplementary Table 4 (noisiness) and 5 (structure clarity).

The results of the qualitative assessment of scan noisiness and structure clarity by the two raters are shown in Supplementary Figure 4. The ordinal mixed models showed that scan lengths of 0.2 and 0.4, as well as head motion measured using mean framewise displacement and mean DVARS, were associated with more noise and less structure clarity on the BOLD delay maps (Figure 6). The quantitative results of the mixed models for noise and structure clarity are shown in Supplementary Tables 4, 5, respectively.

## Discussion

In this study, the effects of scan length on the assessment of brain perfusion using BOLD delay maps in patients with ischemic stroke were systematically investigated. Our results show that scan length can be reduced from 5 min and 40 s to 3 min and 24 s without a significant loss of diagnostic accuracy and image quality of the BOLD delay maps.

A reduction of scan length by nearly two-and-a-half minutes is especially important in acute stroke, where patients are critically ill and decisions have to be made extremely quickly. The standard MRI protocol in our institution takes about 10 min (without perfusion imaging). Because time-to-treatment is a critical factor influencing stroke outcome, prolonging this by anything more than a few minutes is undesirable.

Our findings are overall in accordance with two previous studies. Lv et al. investigated the similarity between areas of BOLD signal delay and areas of hypoperfusion identified by DSC-MRI in acute stroke patients (61). The acquisition time was 5 min 50 s and the scan length was gradually decreased in increments of 10 volumes. They found that BOLD delay maps acquired in 3 min and 4 s provided qualitatively similar information to that of the full length scan in terms of overlap with the mean transit time (MTT; a parameter map derived from DSC-MRI) lesion of the subject. Christen et al., on the other hand, found that BOLD delay maps derived from resting-state functional MRI scans lasting 3 min and 36 s correlated highly with Tmax in Moyamoya patients (23). However, the impact of scan shortening on the diagnostic quality and clinical interpretability of BOLD delay maps was not systematically investigated in these studies.

In this study, agreement between raters on the evaluation of hypoperfusion presence at different scan lengths was good (weighted Cohen's kappa = 0.64 to 0.82). Data on the inter-rater agreement of this relatively new perfusion imaging method has thus far been unavailable. In a study of 105 acute stroke patients, a similar level of inter-rater agreement on detecting perfusion deficits was found for DSC-MRI and ASL, with weighted Cohen's kappa values of 0.64 and 0.6, respectively (62). In our study, agreement on interpretability, structure clarity, and noisiness of the BOLD delay maps was higher for shorter scans than longer scans. This may be explained by the relative ease with which poor quality maps were judged by the raters. Overall, we found that the inter-rater agreement of this relatively new perfusion imaging method is similar to that observed when using more established perfusion imaging methods.

Several factors potentially interact with scan length to influence the diagnostic quality of BOLD delay maps. The temporal resolution of the resting-state functional MRI sequence is one such factor. Although the BOLD oscillations driving the calculation of BOLD delay are likely slow (<0.15 Hz), scanning with high temporal resolution, as done in this study using multiband EPI (42, 43), has the advantage of allowing high-frequency cardiac and respiratory activity to be filtered out of the data.

Head motion, on the other hand, causes undesired changes in the BOLD signal (44, 63) that adversely affect the cross-correlation underlying BOLD delay calculation. In a recent pilot study, we found that the intra-subject reproducibility of BOLD delay values in stroke patients was adversely influenced by head motion (64). In the current study, we therefore accounted for subject head motion in our analysis of the relationship between scan length and BOLD delay map quality and found that head motion significantly and adversely affected the level of noise, the structure clarity, and the volume of BOLD delay lesions, independent of scan length.

Considering that head motion is one of the main drawbacks hindering BOLD delay's use for brain perfusion assessment (14), exploring appropriate and reliable ways of reducing motion and its effects on the BOLD signal is crucial. Retrospective motion correction techniques such as scrubbing, which effectively removes volumes with high motion, have shown promise in functional connectivity studies (44). However, such techniques require that a sufficient amount of low-motion data remain after the removal of high-motion volumes (46). For this to be applicable, we need to know the minimum amount of data needed to generate adequate results. In functional connectivity studies, 10 to 15 min of data generally provide the best test-retest reliability (65). Our results suggest that much less data is required to provide diagnostically acceptable BOLD delay maps. With this knowledge, real-time motion monitoring approaches can allow us to continue scanning until a sufficient amount of low-motion data is acquired (66). This would reduce waste by reducing overscanning and, more importantly, allows scans to be tailored to the urgency of specific clinical situations.

Our study has a few limitations. We were unable to acquire DSC-MRI data in the entire sample in order to directly compare the shortened BOLD delay maps with a reference standard. This was primarily due to the fact that we no longer routinely perform DSC-MRI at our stroke center due to the European Medicines Agency's recent recommendation to restrict the use of gadolinium-based contrast agents (7). However, the relationship between BOLD delay and perfusion measured using DSC-MRI has been established in several independent studies (14, 16, 18–21, 27). In this study, we chose to retrospectively break up longer scans into smaller parts, which is not the same as acquiring several scans of varying lengths. We chose this strategy for practical reasons, as acquiring several scans of different lengths would have greatly prolonged the scanning protocol and would have been infeasible in the context of acute stroke. However, this choice potentially limits how generalizable are results are to real-life situations where shortened scans are independently acquired. In addition, it should be kept in mind that several other potential factors may interact with scan length to influence BOLD delay map quality, including sequence parameters and field strength. Investigating the influence of these factors is beyond the scope of this study, and is currently the focus of ongoing work by our group (64). Finally, whether scan times longer than the full scan implemented in the current study (5 min 40 s) provide even better BOLD delay map quality is yet to be investigated. Such scans would not, however, be suitable in situations where urgent decision-making is required, such as acute stroke.

In conclusion, we show that BOLD delay maps derived from resting-state fMRI scans lasting 3 min 24 s provide sufficient diagnostic quality and adequate assessment of perfusion lesion volumes. This implies that scans can be shortened beyond currently usual scan times, which may be helpful for reducing the effect of patient motion or in situations where quick clinical decisions need to be made. Our results represent an important step toward implementing BOLD delay for contrast-agent-free assessment of brain perfusion in acute stroke patients in routine clinical practice.

## Data Availability Statement

The data and the code used for statistical analysis and data visualization in this study are publicly available at https://github.com/ahmedaak/BD_scan-shortening.

## Ethics Statement

The studies involving human participants were reviewed and approved by Charité institutional review board EA1/200/13. The patients/participants provided their written informed consent to participate in this study.

## Author Contributions

AT, AK, JF, and AV: Conception or design of the work. JF, KV, IG, EK, and AK: Data collection. UG, IG, KV, AK, and AT: Data analysis and interpretation. AT and AK: Drafting the article. AT, KV, IG, UG, EK, JF, AV, and AK: Critical revision of the article and final approval of the version to be published.

## Conflict of Interest

KV and JF were funded by the German Federal Ministry of Education and Research (01EO0801, 01EO01301). JF has received consulting, lecture, advisory board fees from BioClinica, Cerevast, Artemida, Brainomix. AK, KV, and JF were co-inventors of a patent application relating to a method for automated, user-independent delineation of perfusion lesions, used in this manuscript. The remaining authors declare that the research was conducted in the absence of any commercial or financial relationships that could be construed as a potential conflict of interest.
