# Supplementary material for: The Effect of Scan Length on the Assessment of BOLD Delay in Ischemic Stroke
Source: Front Neurol. 2020 May 5;11:381. doi: 10.3389/fneur.2020.00381 (PMC7214917; doi:10.3389/fneur.2020.00381)
Supplement: Supplementary file 1 [file Data_Sheet_1.pdf]

Supplementary Table 1 - Results of the linear mixed model of BOLD delay lesion volume.

Category	*Predictor*	*Coefficient (beta)*	*95% CI of coefficient*	*t-value*	*p-value*
**(Intercept)**		**2.26**	**1.42 - 3.09**	**5.31**	**<0.0001**
Scan length	0.8	0.01	-0.16 - 0.17	0.10	0.9186
	0.6	0.08	-0.09 - 0.24	0.90	0.3698
	0.4	0.02	-0.15 - 0.19	0.25	0.8042
	0.2	-0.11	-0.28 - 0.06	-1.31	0.1927
	Mean FD	0.12	-0.004 - 0.24	1.88	0.0616
	**Mean DVARS**	**0.05**	**0.02 - 0.07**	**3.64**	**0.0003**
Session	**Day 1**	**-0.22**	**-0.39 - -0.05**	**-2.47**	**0.0139**
Threshold	**2.3s**	**-0.93**	**-1.06 - -0.80**	**-14.13**	**<0.0001**
	**4.6s**	**-1.58**	**-1.71 - -1.46**	**-24.02**	**<0.0001**

FD: framewise displacement. The reference levels for the categorical variables scan length, session, and threshold were full scan, day 0 (baseline), and 0s respectively. FD: framewise displacement, DVARS: the frame-to-frame root mean square change in voxel intensities averaged across the entire brain.

Supplementary Table 2 - Agreement (unweighted Cohen's kappa) between BOLD delay maps of different scan lengths and the BOLD delay maps derived from the full scan

Scan length	*Rater 1*	*Rater 2*
**0.8**	0.67	0.58
**0.6**	0.54	0.49
**0.4**	0.41	0.44
**0.2**	0.24	0.30

Supplementary Table 3 - Results of the binary logistic mixed model of BOLD delay map interpretability.

Category	*Predictor*	*Odds ratio*	*95% CI*	*p-value*
Scan length	0.8	1.06	0.58 - 1.93	0.8593
	0.6	0.93	0.51 - 1.70	0.7993
	**0.4**	**0.37**	**0.21 - 0.64**	**0.0004**
	**0.2**	**0.21**	**0.12 - 0.37**	**<0.0001**
	Mean FD	1.28	0.90 - 1.80	0.1679
	Mean DVARS	0.96	0.90 - 1.02	0.1429
Session	**Day 1**	**1.93**	**1.27 - 2.92**	**0.0019**
Rater	**Rater 2**	**0.57**	**0.40 - 0.81**	**0.0016**

FD: framewise displacement, DVARS: the frame-to-frame root mean square change in voxel intensities averaged across the entire brain.

Supplementary Table 4 - Results of the ordinal mixed model of BOLD delay map noise.

Category	*Predictor*	*Odds ratio*	*95% CI*	*p-value*
Scan length	0.8	1.11	0.73 - 1.69	0.6392
	0.6	1.20	0.79 - 1.83	0.4001
	**0.4**	**3.60**	**2.32 - 5.58**	**<0.0001**
	**0.2**	**6.84**	**4.30 - 10.9**	**<0.0001**
	**Mean FD**	**1.80**	**1.13 - 2.86**	**0.0131**
	**Mean DVARS**	**1.07**	**1.01 - 1.13**	**0.0196**
Session	**Day 1**	**0.68**	**0.49 - 0.94**	**0.0197**
Rater	**Rater 2**	**2.03**	**1.54 - 2.67**	**<0.0001**

FD: framewise displacement, DVARS: the frame-to-frame root mean square change in voxel intensities averaged across the entire brain.

Supplementary Table 5 - Results of the ordinal mixed model of BOLD delay map structure clarity.

Category	*Predictor*	*Odds ratio*	*95% CI*	*p-value*
Scan length	0.8	1.17	0.77 - 1.76	0.4663
	0.6	1.32	0.87 - 1.99	0.1886
	**0.4**	**3.09**	**2.03 - 4.71**	**<0.0001**
	**0.2**	**6.93**	**4.44 - 10.80**	**<0.0001**
	**Mean FD**	**1.83**	**1.23 - 2.72**	**0.0030**
	**Mean DVARS**	**1.06**	**1.01 - 1.11**	**0.0175**
Session	**Day 1**	**0.61**	**0.44 - 0.83**	**0.0021**
Rater	**Rater 2**	**2.46**	**1.88 - 3.22**	**<0.0001**

FD: framewise displacement, DVARS: the frame-to-frame root mean square change in voxel intensities averaged across the entire brain.

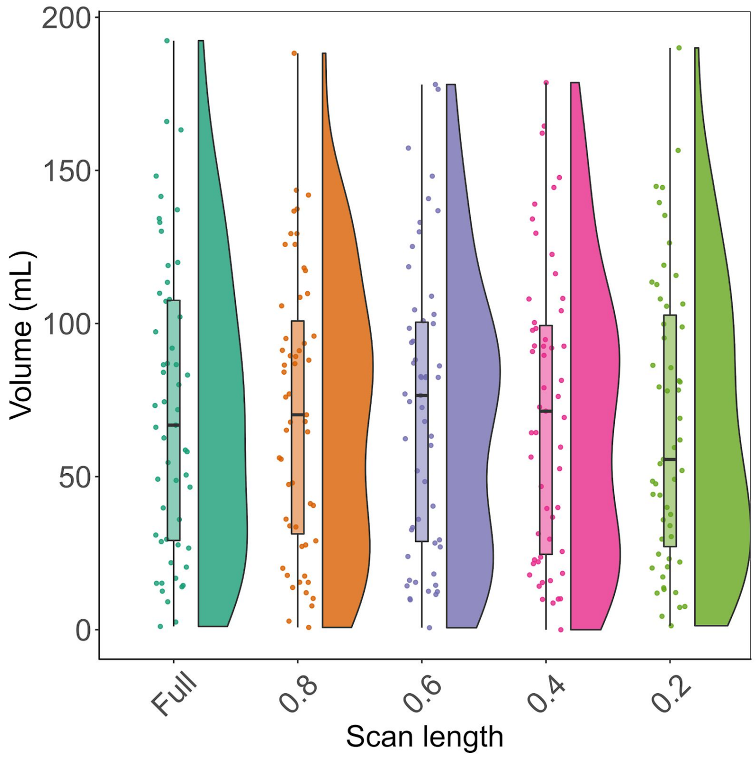

Supplementary Figure 1. Raincloud plots showing the distribution of BOLD delay lesion volumes derived from resting-state functional MRI scans of different lengths.The quantitative results of the linear mixed model investigating the effects of scan length on BOLD delay lesion volumes are shown in Supplementary Table 1.

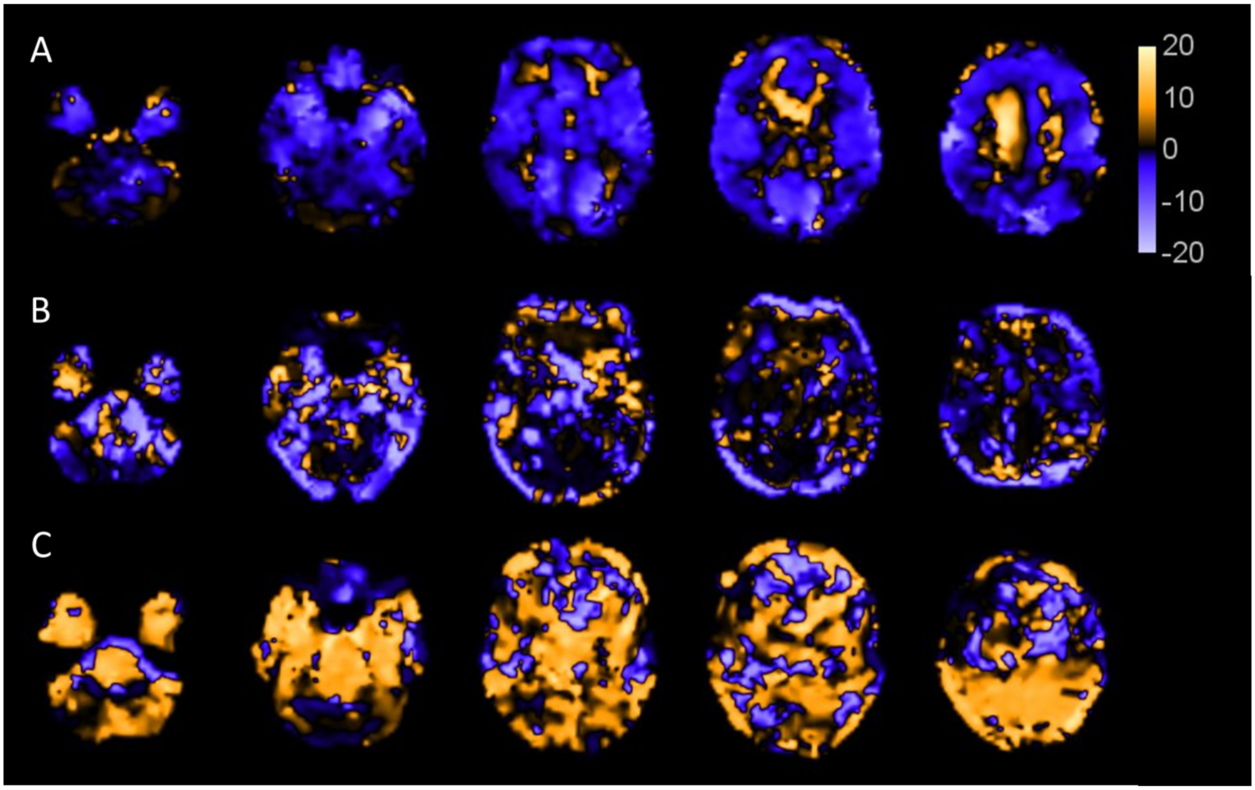

Supplementary Figure 2. Example BOLD delay maps of different quality. Row A shows a map with low noisiness (relatively little BOLD delay in the brain parenchyma) and high structure clarity (symmetrical BOLD delay within the ventricles and periventricular white matter). Row B shows a map with medium noisiness - note the scattered, symmetrical areas of BOLD delay within the brain parenchyma. Row C shows a map with high noisiness, showing large, confluent, symmetrical areas of BOLD delay within the brain parenchyma. Note that asymmetrical areas of BOLD delay occurring in a pattern consistent with the brain's vascular territory distribution were not considered noise and were assessed by the raters as reflecting stroke-related hypoperfusion.

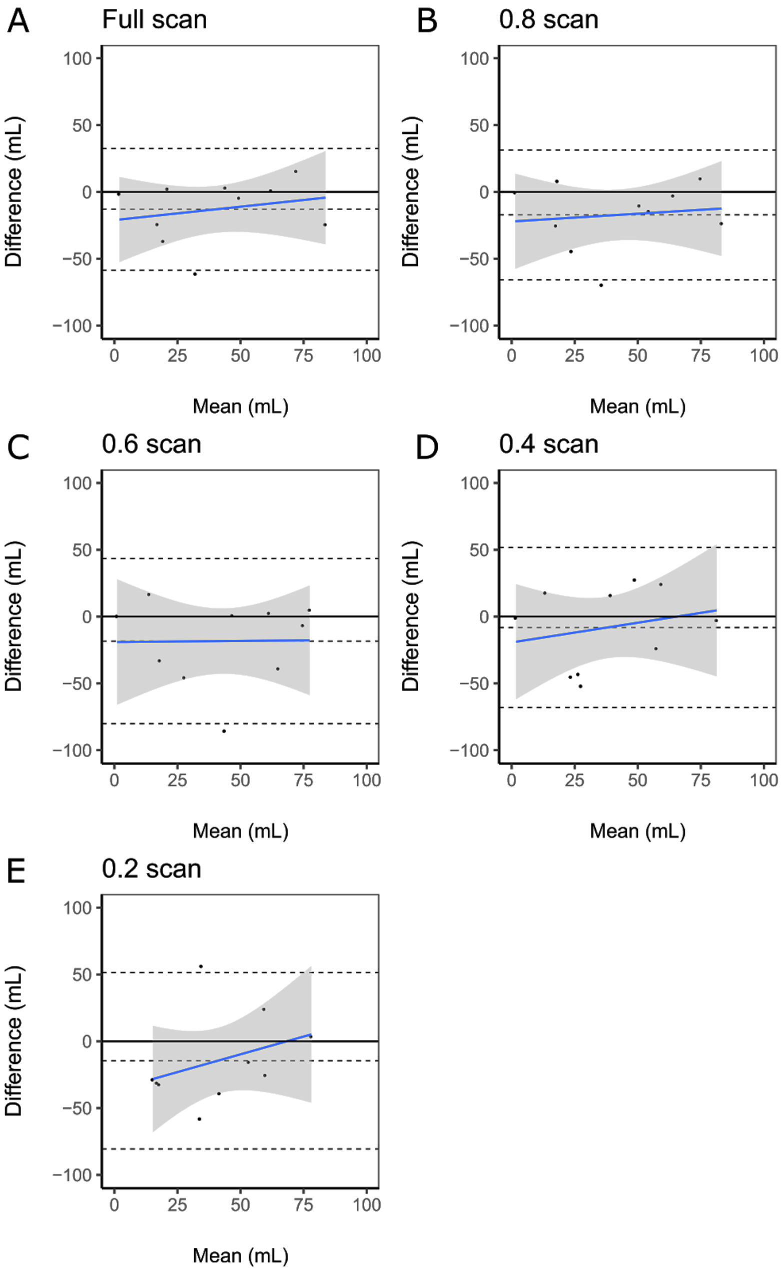

Supplementary Figure 3. Bland-Altman plots of volumetric agreement between BOLD delay lesion volumes derived from resting-state functional MRI scans of different lengths and perfusion lesion volumes derived from Tmax maps. The upper and lower dashed lines represent the 95% limits of agreement and the middle dashed lines represent the bias (mean difference). The blue solid line and shaded grey region represent the regression lines and 95% confidence interval of the regression lines respectively.

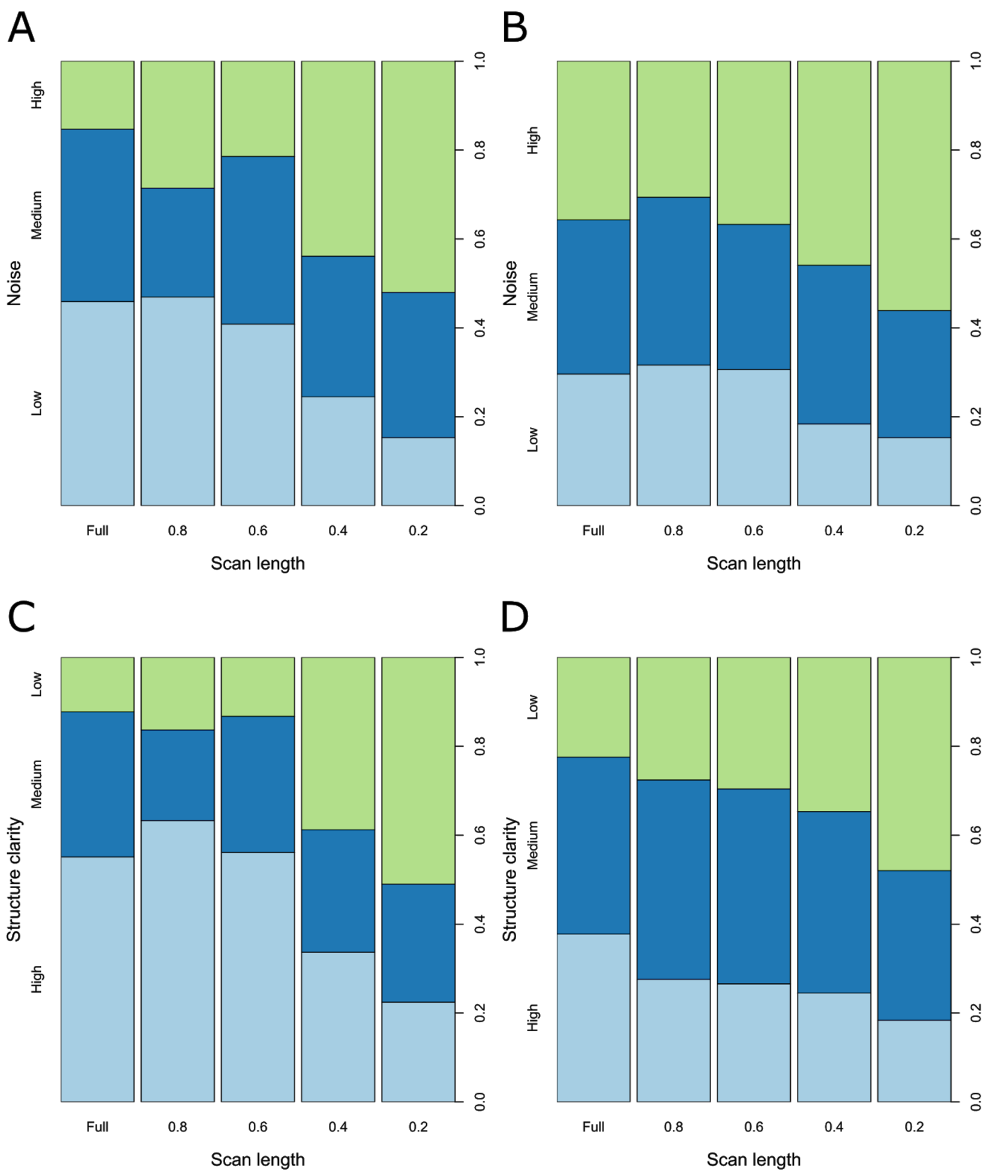

Supplementary Figure 4. Spine plots showing the distribution of the qualitative ratings of the BOLD delay maps derived from different resting-state functional MRI scan lengths made by rater 1 (A and C) and 2 (B and D). Figures A and B show the ratings of map noisiness, while Figures C and D show the ratings of structure clarity. The quantitative results of the ordinal mixed model investigating the effects of scan length on the noisiness and structure clarity of the BOLD delay maps are shown in Supplementary Tables 4 and 5 respectively.
